# Safety and Efficacy of Percutaneous Nephrolithotomy in Solitary Functioning Kidneys: A Retrospective Cohort Study in an Asian Population

**DOI:** 10.7759/cureus.55728

**Published:** 2024-03-07

**Authors:** Muhammad Shoaib Mithani, Wajahat Fareed, Neha Asif, Mishquat Shabbir

**Affiliations:** 1 Urology, The Kidney Centre Postgraduate Training Institute, Karachi, PAK; 2 Urology, Jinnah Sindh Medical University, Karachi, PAK

**Keywords:** single renal unit, kidney stone disease, single kidney, solitary kidney, percutaneous nephrolithotomy (pcnl)

## Abstract

Objective

The aim of the study was to assess the safety of preserved renal function after standard percutaneous nephrolithotomy (PCNL) in patients with a single functional kidney. The main parameters to focus on were serum creatinine levels and any associated complications.

Materials and methods

This retrospective cohort study was conducted in an Eastern population in a single center from 2016 through 2023 at The Kidney Centre Postgraduate Training Institute, Karachi, Pakistan.

Results

Out of the total 1,550 PCNL procedures performed on adult patients, 47 patients had a solitary functioning kidney with stones, which were evaluated. The stone clearance rate was 95.74% (45 patients), with a mean operative time of 85.96 minutes. Most patients, i.e., 33 (70.21%), had an infracostal approach, and single tract management was sufficient for 45 (95.74%) patients. The most common complication was transfusion, which was required in five (10.64%) patients. Mean preoperative hemoglobin dropped by 1.43mg/dL postoperatively, and mean serum creatinine decreased from 2.45mg/dL to 1.97mg/dL. Among the 24 (51.06%) analyzed stones, all were calcium oxalate.

Conclusion

In challenging situations such as a solitary kidney with a large stone, PCNL is the procedure of choice. However, the refined technique is of paramount importance. Overall, the use of PCNL in these unique conditions is safe and rewarding.

## Introduction

Renal stones are a major health burden in urological practice, especially in the working age group, and there is an increasing trend noticed in its incidence [1]. The prevalence has increased around two to four times in the last 40 years [2]. Around 12% of men develop urolithiasis in their lifetime compared to 6% of women [3]. Overall, 2-5% of people suffer from symptomatic renal stones in their lifetime [4].

Treating renal stones can be challenging in patients with solitary kidneys. Multiple treatment options are available for the treatment of renal stones, such as retrograde intrarenal surgery, extracorporeal shock wave lithotripsy, and percutaneous nephrolithotomy (PCNL) [5]. PCNL has been the gold standard treatment option for complex renal stones. PCNL has been associated with major complications, such as pleural effusion, bleeding, pneumothorax, infection, residual stones, and compromised renal functions, which can be devastating for patients with a compromised renal function as is likely in cases of solitary functioning kidney [6-8]. The surgical technique needs to be meticulous in these patients to preserve renal function as much as possible and to provide good stone clearance.

There is evidence in the literature that PCNLs performed in solitary functioning kidneys have been associated with a greater complication rate as compared to bilaterally functioning kidneys [9]. However, much of the data come from the Western population. This study aims to study the safety and efficacy of PCNL in solitary functioning kidneys treated in a stone center in an eastern population. This is the first study addressing this complex phenomenon in an institution in Pakistan.

## Materials and methods

Study design and methodology

This was a retrospective cohort study. After obtaining approval from the Ethical Review Board of our institute, the study included all patients who underwent PCNL in The Kidney Centre between 2016 and 2023, who had solitary functioning or anatomically solitary kidney, and were between 17 and 80 years of age. All the patients who were selected had complete laboratory workups available including the baseline labs. Patients also had radiological workup available, including preoperative CT scan, X-ray, and/or ultrasound to assess the size, characteristics, and location of stones, as well as the anatomy of the kidney. All the patients had undergone PCNL in a prone position by experienced surgeons with over 15 years of experience. After anesthesia induction, retrograde pyelogram (RPG) was performed in all patients in lithotomy position to study the pelvicalyceal system and confirm the location of stones. A retrograde open-ended 5-Fr ureteric catheter was placed in the ipsilateral kidney and secured with a Foley catheter. The position was then changed to prone. The procedure involved a renal puncture that was guided by fluoroscopy, utilizing an 18-gauge needle. The approach, either supracostal or infracostal, was chosen based on the location of the stones and the anatomy of the kidney. A guidewire was passed through the needle, and the tract was dilated first with fascial dilators and then with telescopic metallic dilators up to 27 Fr. Then a 30-Fr Amplatz sheath was placed. A 26-Fr nephroscope was also used. In very few cases in which the stone sizes were smaller than 2cm, mini-nephroscopes were used. Stones were fragmented using the pneumatic lithoclast (KARL STORZ, Tuttlingen, Germany), and fragments were removed with forceps. Fluoroscopic clearance was recorded. Postoperatively, a double J stent and/or nephrostomy tube was placed when indicated. Postoperatively, patients underwent X-ray or ultrasound on the first postoperative day to assess the stone-free rate (SFR) and size of residual stones, if any. Hemoglobin and creatinine levels of the patients were also checked on the first postoperative day. Patients who had deranged creatinine levels were checked for creatinine levels on follow-up in the OPD six weeks after the procedure to look for the long-term impact of PCNL on renal function. Patients who had raised creatinine levels were considered as subjects for the subsequent study. All the patients who had bilateral functioning kidneys, those with a history of renal transplant, and those with incomplete medical records were excluded from this study.

Data collection

The patients were selected from the above-mentioned criteria. Medical records and investigations of those patients were retrieved. The patient’s characteristics and investigations were assessed. Data were collected on questionnaire forms. Demographic information (including age, gender, and comorbidities), preoperative imaging (for stone size and location), intraoperative details (such as duration, approach, and complications), postoperative outcomes (including clearance rate, complication, and hospital stay), and pre- and postoperative laboratory parameters were all recorded. Data were entered into SPSS software Version 27 (IBM Corp., Armonk, NY).

Data analysis

Data were analyzed using SPSS software Version 27. Qualitative data (gender, SFR, etc.) were documented using the number and the percentage of the whole, while quantitative data (age, stone density, stone size, etc.) were expressed in mean and standard deviation. The results were compared with those in recent literature and further elaborated on in the discussion.

## Results

**Figure 1:**
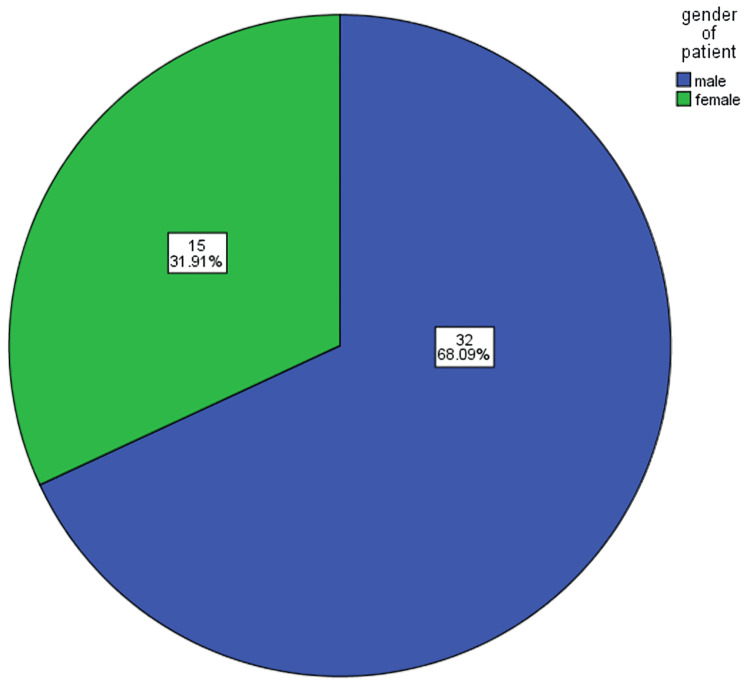
Gender distribution of the studied patients

A total of 47 patients were assessed, out of which 32 (68.09%) were males and 15 (31.91%) were females (Figure 1). Of these 47 cases, 31 (65.97%) patients had their kidney removed due to renal stones in the past. Three (6.38%) had secondary to missed pelviureteric junction obstructions, and the remaining 14 (29.79%) patients had a contralateral atrophic non-functioning kidney after having an open procedure in the past.

**Figure 2:**
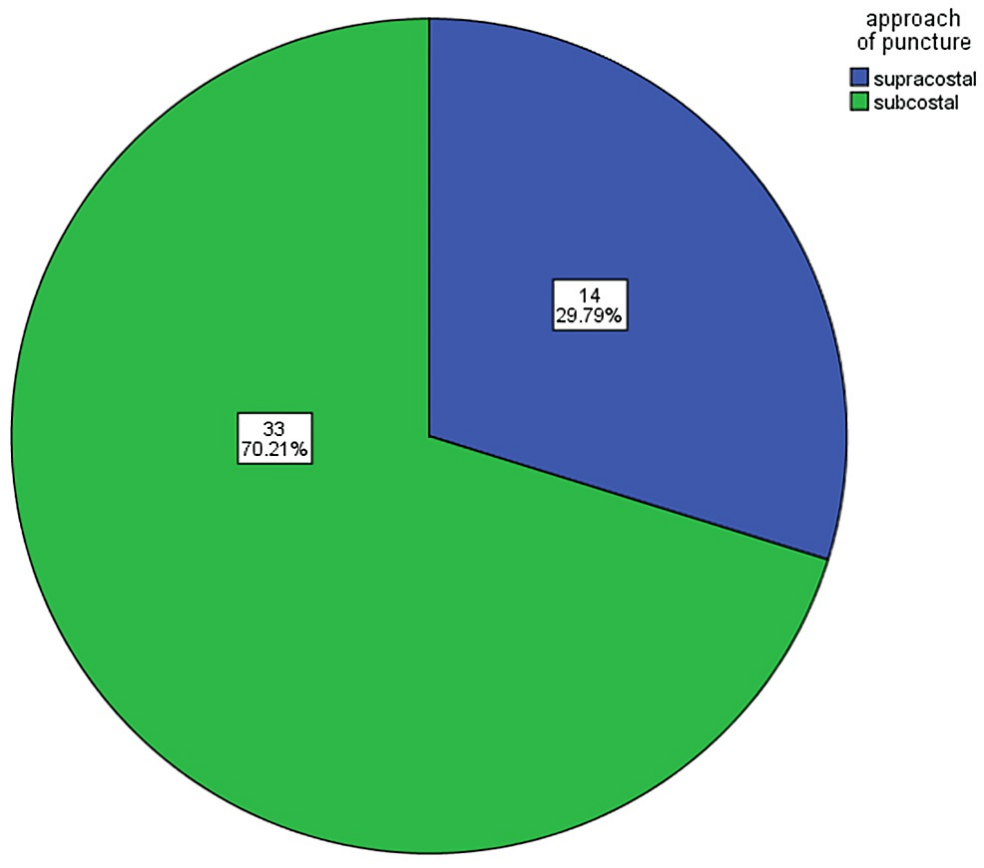
Puncture site

**Figure 3:**
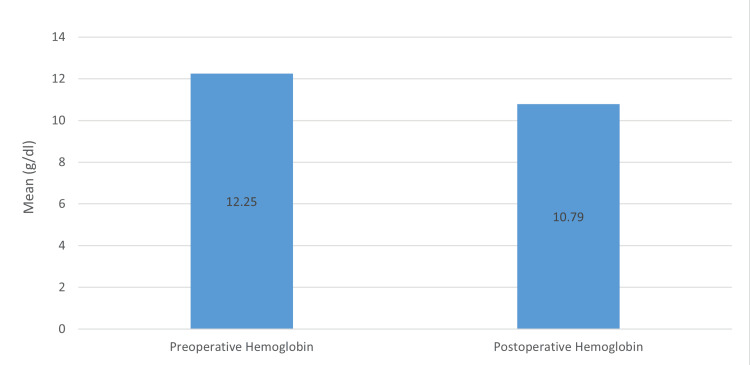
Change in the hemoglobin level

**Figure 4:**
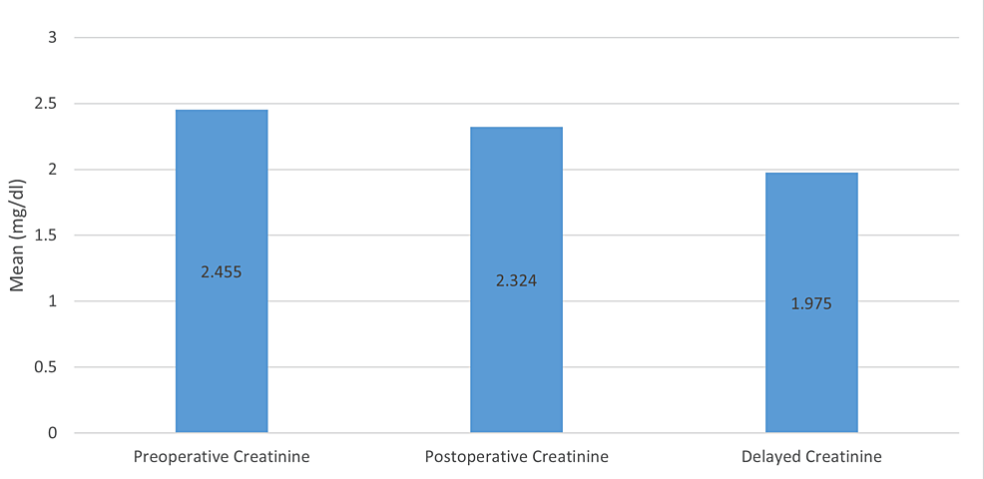
Change in the creatinine level

The mean age was 39.89 ± 15.33 years (Table 1). The average stone size was 2.8cm (Table 1), ranging from a minimum of 1.8cm and a maximum of 3.2cm. The mean operative time was 85.96 minutes (Table 1). Stone clearance rate was 95.74% (45 patients), taking 4mm as a cut-off (Table 2). Overall, 14 patients (29.79%) had a supracostal approach and the rest of the 33 patients (70.21%) had an infracostal approach (Figure 2). Only two patients (4.26%) required multiple tracts, while the rest of the patients were managed by single tract (Table 2); also, 27 (57.45%) patients had their superior calyx punctured, 19 (40.43%) patients had their lower calyx punctured, and one (2.13%) patients were dealt with middle calyx puncture. The most common complication reported was the requirement of transfusion, which occurred in five (10.64%) patients. Other complications reported were as follows: postoperative fever in three (6.38%) patients, urosepsis in two (4.26%) patients, pleural effusion in one (2.13%) patients, and failed access in one (2.13%) patients (Table 2). Mean preoperative hemoglobin was reported to be 12.25mg/dL, while mean postoperative hemoglobin was 10.79mg/dL with a drop of 1.43mg/dL (Figure 3). Mean serum creatinine at baseline was 2.45mg/dL, which decreased to 2.32mg/dL postoperatively. When patients were further followed, they had a mean serum creatinine level of 1.97mg/dL with a decrease of 0.48mg/dL from baseline (Figure 4). Among the 24 (51.06%) patients who had their stone analysis conducted, all of them had calcium oxalate stones.

**Table 1:** Patient characteristics

Characteristics	Mean
Weight	62.56 kg
Age	39.89 years
Male/female ratio (%)	68/32
Stone size (cm)	2.8255 cm
Operative time incl. induction, positioning, procedure, and extubation (minutes)	85.96 minutes

**Table 2:** Procedure-related parameters PCNL, percutaneous nephrolithotomy

Complications	Percentage
Transfusion	10.64% (n = 5)
Failed access	2.13% (n = 1)
Effusion	2.13% (n = 1)
Chest intubation	0.00% (n = 0)
Postop fever	6.38% (n = 3)
Urosepsis	4.26% (n = 2)
Re-PCNL	0.00% (n = 0)
Stone clearance	95.74% (n = 45)
Single tract	95.74% (n = 45)
Double tract	4.26% (n = 45)
Change in hemoglobin	-1.46mg/dL
Change in serum creatinine	0.13mg/dL

## Discussion

Renal stone management in patients with a solitary kidney is a complex task that requires careful consideration of various factors. This discussion incorporates findings from relevant studies to provide a comprehensive understanding of the challenges and outcomes associated with PCNL in this unique patient population.

The investigation by Jones et al., which specifically addressed PCNL and its application in patients with a solitary kidney, emphasized the importance of this intervention in the unique context of a solitary kidney. PCNL alone resulted in a 77.3% SFR or fragments of 2mm or less. Still, patients had a previously operated kidney in 35.8% of cases, and 52.9% of patients had complete or partial staghorn calculi and 18.8% had multiple stones [10]. In our case, the SFR was 95.74% (45 patients), but the cut-off for stone clearance was defined as 4mm or less. A 4mm cut-off for the stone clearance was deemed acceptable because stones of this size generally have a higher likelihood of spontaneous passage or can be managed with less invasive interventions, reducing the need for additional surgical procedures. Stone clearance was assessed on the first postoperative day with an X-ray of the kidney, ureter, and bladder in cases in which the stone was radiopaque and with ultrasonography in cases in which the stone was radiolucent. Furthermore, Ruiz Marcellan et al. provided valuable insights into the treatment of urolithiasis in individuals with a solitary kidney, offering additional perspectives on the efficacy and considerations associated with managing renal stones in this specialized population. In this study, initial SFR and final SFR were 72.7% and 100%, respectively [11]. Both studies contribute to the broader understanding of the role of PCNL in addressing lithiasis within the challenging framework of solitary kidneys, highlighting its significance in clinical practice.

Concerns surrounding the impact of PCNL on renal function in solitary kidneys have been extensively addressed in the literature, drawing insights from various studies. Modern management practices for stone disease in such cases have been explored, emphasizing the need to minimize risk factors [12,13]. A valuable perspective is provided by a prospective, observational study that examines perioperative renal functional changes among patients with larger renal stones, offering nuanced insights into PCNL outcomes. In this study, they used ultrasound-guided puncture to reduce morbidity and decline in renal function. They reported a significant improvement in estimated glomerular filtration rate from 53.9 ± 24.0 to 61.3 ± 25.4 mL/min/1.73 m^2^ (P < 0.01). Renal function was stable, improved, and worse in 65.9% (n = 29), 27.3% (n = 12), and 6.8% (n = 3) of patients, respectively, compared with preoperative levels [14]. Furthermore, the investigation into the effects of multiple access tracts on renal function underscores the importance of a meticulous approach to mitigate complications that may compromise renal function [15]. In our study, only two (4.26%) patients required multiple tracts. The transfusion rate of 10.64% (five patients) in patients with solitary kidneys in our study is noteworthy, as it falls within the reported range of transfusion rates in similar populations [16]. This emphasizes the importance of vigilant perioperative management to minimize the risk of complications, particularly in individuals with compromised renal function. Together, these references contribute to a comprehensive understanding of the multifaceted impact of PCNL on renal function, particularly in the context of solitary kidneys.

The mean serum creatinine of 2.25, with a postoperative change of 0.13mg/dL, reflects a relatively stable renal function following PCNL. While any change in serum creatinine warrants attention, the modest increase observed suggests a judicious approach to surgical intervention.

Complications associated with PCNL in solitary kidneys have been investigated, shedding light on the safety profile of the procedure [17,18]. Studies have assessed factors influencing complications, such as the size of renal calculi and the presence of underlying renal insufficiency. Kuzgunbay et al. conducted a study to assess the impact of PCNL on long-term outcomes and stone recurrence in patients who already had compromised renal function. The mean serum creatinine value was 2.30 ± 0.56mg/dL before surgery and 2.67 ± 1.41mg/dL at the end of follow-up (p = 0.386). Also, 12.5% of patients had recurrence after the follow-up of 51 months, which can have a great impact on patients’ quality of life considering already compromised renal function and the need for repeated interventions [19].

These results prompt considerations for refining surgical techniques, perioperative care, and patient selection to enhance the safety of PCNL in solitary functioning kidneys. The findings contribute to the growing body of evidence on renal stone management, particularly in populations where data is sparse.

The reason behind using creatinine levels for estimating the residual renal function over quantitative renal scans is that the latter may not be ideal for assessing a solitary kidney’s function post-surgery, especially when creatinine levels are high. These scans are better suited for evaluating the comparative function of two kidneys. Instead, the glomerular filtration rate, estimated through serum creatinine and factors such as age and sex, offers a more precise measure of kidney function. For patients with one kidney removed and the other treated, renal scans are unnecessary for determining side-specific function; hence, their exclusion from data variables supports consistency.

Limitations

Despite the valuable insights gained, this study has limitations. The study was conducted in the retrospection design, and the sample size, although reflective of the study population, may limit generalizability. Additionally, variations in surgical expertise and patient characteristics could influence outcomes. (Data analyzed did not differentiate between previously operated kidneys and those that were operated for the first time.)

## Conclusions

This study provides valuable insights into the safety and efficacy of PCNL in solitary functioning kidneys, specifically within an eastern population. The observed stone clearance rates and complication rates contribute to the evolving understanding of PCNL outcomes in this challenging patient subgroup. Future prospective studies with larger cohorts and longer follow-up periods are warranted to further refine clinical practices and improve outcomes in this specialized field of urological practice.
